# Non O1/ O139 Vibrio *Cholerae Septicemia* in a Patient with Hepatocellular Carcinoma

**DOI:** 10.4314/ejhs.v31i6.27

**Published:** 2021-11

**Authors:** Arun Sachu, Deepak Johnson, Sabu Thomas, Renu Mathew

**Affiliations:** 1 Assistant Professor, Department of Microbiolog, Believers Church Medical College, Thiruvalla, Kerala; 2 Consultant Gastroenterologist, Believers Church Medical College, Thiruvalla, Kerala; 3 Faculty Scientist and Principal Investigator, Cholera and Biofilm Research Laboratory, Rajiv Gandhi Centre of Biotechnology; 4 Department of Microbiology, Believers Church Medical College, Thiruvalla, Kerala

## Abstract

BACKGROUND: An elderly male with underlying Hepatocellular carcinoma came with history of fall with head and ear trauma, vomiting, abdominal pain, fatigue. Patient died within hours due to Septic shock Blood culture grew Non O1/O139 Vibrio cholerae which was later found to be non-toxigenic. This was a lethal case of non-O1/non-O139 V. cholerae sepsis and we focus on the dilemmas in identification and management of this rare bacterium.

## Introduction

Although over 200 serogroups of *Vibrio cholerae* have been identified so far, there are only two serogroups (O1 and O139) which are the common cause of epidemic cholera. NOVC(“non-O1/O139 *V. cholerae*”)strains are generally nonpathogenic but there have been reports of intestinal and extra-intestinal infections caused by NOVC strains (1). The invasive infections caused by NOVC strains have a fatal outcome when compared to the gastrointestinal infections which are less severe. These strains can cause sepsis in immunodeficient hosts such as those with cirrhosis and hematological malignancies. Hypothesis suggested for the increased incidence of sepsis in cirrhotic patients include reduced serum bactericidal activity, cirrhotic liver with poor filtration function and raised serum iron levels. Our report is a lethal case of Non O1 /O139 strain causing sepsis in a patient with Hepatocellular carcinoma. Informed consent was taken from the patient's relatives.

## Case Summary

An elderly male diagnosed with alcohol induced cirrhosis with HCC(Hepatocellular carcinoma)came to the emergency department with history of fall with head and ear trauma, vomiting, abdominal pain, fatigue. There was no history of diarrhoea. He was previously declared Hepatitis B positive. He was posted for Liver Transplant in another hospital.

On clinical examination, patient was drowsy. Pallor and icterus were present. The patient's temperature was 38 °c, Hb- 11.2g/dl, PR-86 /mt, BP- 130/80 mm Hg. Laboratory Investigations are shown in Table 1.

**Table 1:** Laboratory Investigations

Parameters	Value	Normal Range
Procalcitonin	**85.34**	0.1–0.49ng/ml
Total Bilirubin	6.94	0.2–1 mg/dl
SGOT (Aspartate aminotransferase)	86	10–40U/L
SGPT (Alanine transaminase)	52	10–40U/L
Alkaline Phosphatase	246	30–120 U/L
Lactate	10.4	0–1mmol/L
Total Count	7000	4000–11,000cells/microlitre
Neutrophil Count	1000	1500–8000 cells/microlitre

ng/ml-nanogram/millilitre, mg/dl- milligram/decilitre, U/L- Units/litre

Blood was drawn into two aerobic and two anaerobic bottles for culture. Antibiotic therapy was initiated with meropenem. Elevated Procalcitonin levels was indicative of septic shock. The condition of the patient deteriorated within 5 hrs of hospitalization and he died of septic shock. Blood culture bottles were loaded into the automated culture system (Bact T/ALERT/ 3D; Biomerieux, Marcy L Etoile, France) which is available in the laboratory. Aerobic and anaerobic blood culture bottles became positive after 8hrs of incubation and Gram stain from the blood culture broth revealed gram negative bacilli. Blood culture broth was inoculated into Blood and MacConkeys agar (Himedia Laboratories, Pvt. Ltd, Mumbai, India) and incubated.

And, 5% sheep blood agar grew beta-haemolytic mucoid colonies, Haemodigestion was clearly seen (Figure 1). MacConkeys agar grew non lactose fermenting colonies. The colonies were oxidase positive and gram stain from the growth showed curved gram negative bacilli. VITEK 2 compact system(BioMérieux, France)gave an identification of *V. cholerae* with 98% probability and an “Excellent identification” confidence level. Thiosulfate-citrate-bile salt-sucrose agar gave characteristic yellow-coloured colonies of *V. cholerae*. Cholera red reaction (reddish pink colour in the presence of peptone water due to the formation of nitrosoindole) was positive. Organism failed to agglutinate with O1 and O139 antisera. The isolate was found to be susceptible to ampicillin, piperacillin, cephalosporins, aminoglycosides, flouroquinolones, tetracycline, chloramphenicol and trimethoprim sulfamethoxazole. Antibiotic discs were obtained from Himedia (Himedia Laboratories, Pvt. Ltd, Mumbai, India). CLSI(Clinical Laboratory and Standards Institute) Criteria for fastidious bacteria was used and antibiotic susceptibility was performed on Muller Hinton Agar using Kirby Baeur disc diffusion method. Identification of the organism was further confirmed as *Vibrio cholerae* by MALDI-TOF MS(BioMérieux, France).

The identity of the organism was finally confirmed by PCR amplification of V. cholerae species-specific conserved intergenic spacer region between 16S and 23S rRNA. Further analysis of the isolate at Rajiv Gandhi Centre for Biotechnology (RGCB), Trivandrum revealed that the isolate did not amplify the ctx A(cholera toxin A) and ctx B(cholera toxin B) genes that code for the cholera toxin A and B subunit which indicated it to be non-toxigenic. The isolate showed agglutination with 0–110 antisera when the serogrouping of the isolate was carried out by a reference laboratory (National Institute of Cholera and Enteric Diseases, Kolkata, India).

## Discussion

NOVC is a curved gram negative bacterium, generally considered to be non-pathogenic but can cause diarrheal illness, wound infection, and fatal septicemia in immunocompromised hosts (Table 2) (2,3). This case showed the lethal effects of an exceedingly rare organism on an immunosuppressed host.

**Table 2:** Case reports from India of “non o1/o139” Vibrio cholerae causing septicemia

Author	Clinical presentation	Mode of transmission	Outcome
Chowdhury et al^2^	Abdominal pain	Unknown	Died
Chowdhury et al^2^	Swelling of right leg	Unknown	Died
Chowdhury et al^2^	Fever, chills	Unknown	Recovered
Khan et al^3^	Fever, abdominal distension	Unknown	Died
Current case report	Abdominal pain, Vomiting	Unknown	Died

Infection with rare bacterium is mainly seen among middle-aged males and rarely among children <18 years old. The source of NOVC bacteraemia includes seafood consumption, drinking contaminated water and contamination by direct invasion through grazed skin or wounds. The main clinical presentations of NOVC infections include mild to severe gastroenteritis, wound and ear infections and fatal bacteremia. The bacteremia which is usually fatal presents with hypo or hyperthermia, mostly watery diarrhoea, abdominal pain, chills, hypotension, nausea and vomiting.

Source of infection in our patient could not be pinpointed but since the patient came from an area where backwaters are common source of drinking water, it is quite possible that the patient was infected by an environmental strain. Eventhough the prognosis of gastroenteritis due to NOVC is generally excellent, case fatality rate of NOVC bacteremia among cirrhotic patients ranges from 23.8% to 61.5% (4). The reason for the virulence of NOVC among immunocompromised hosts maybe attributed to the haemolytic property of the organism. Our isolate lacked the virulence genes encoding for CT (cholera toxin), Stn (heatstable enterotoxin), Chx(cholix toxin).

Microbiology laboratories face a lot of dilemmas in confirming infections due to NOVC mainly due to the fact that the antisera are only available in very few centres. Molecular methods are not available in most laboratories. The time taken for the patient to present to the hospital along with immunocompromised state of the individual probably contributed to the death of the patient, eventhough the organism grown in culture was multisensitive. Literature has shown that for NOVC bacteremia,various antimicrobial regimens like cefotaxim, minocycline, tetracycline and fluoroquinolones can be used(5).

In conclusion, there is an urgent need to strengthen the capacity of microbiology laboratories throughout the country by ensuring the availability of required antisera which will result in prompt diagnosis of Noncleaving to better patient care. Physicians should be aware of these infections, so that they educate the patients especially those presenting with risk factors for bacteremia, on the importance of avoiding contact with seawater or fresh water and also to educate them about the risks of consuming raw or undercooked seafood.
